# Alisol C 23-acetate from the rhizome of *Alisma orientale*

**DOI:** 10.1107/S1600536808032959

**Published:** 2008-10-31

**Authors:** Zha-Jun Zhan, Hong-Ling Bian, Wei-Guang Shan

**Affiliations:** a College of Pharmaceutical Science, Zhejiang University of Technology, Hangzhou 310014, People’s Republic of China

## Abstract

The title compound [systematic name: 11β-hydr­oxy-24,25-ep­oxy-3,16-oxo-protost-13 (17)-en-23-yl acetate], C_32_H_48_O_6_, a protostane-type triterpenoid, was isolated from the Chinese herbal medicine alismatis rhizoma (the rhizome of *Alisma orientalis* Juzep). The mol­ecule contains four *trans*-fused rings, *viz.* three six-membered and one five-membered ring. Two of the six-membered rings have slightly distorted half-chair conformations, while the third exhibits a chair conformation. The five-membered ring is almost planar. An inter­molecular O—H⋯O hydrogen bond between the hydr­oxy and ep­oxy groups and intra- and intermolecular C—H⋯O hydrogen bonds are observed.

## Related literature

For related literature, see: Nakajima *et al.* (1994 ▶); Yoshikawa *et al.* (1993 ▶, 1997 ▶). 
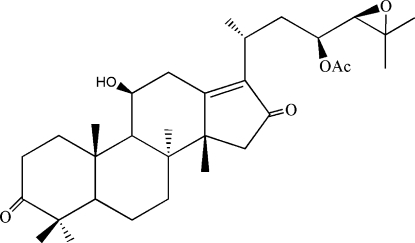


## Experimental

### 

#### Crystal data

C_32_H_48_O_6_
*M* *_r_* = 528.73
Orthorhombic, 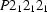

*a* = 7.6879 (3) Å
*b* = 14.6555 (6) Å
*c* = 26.9557 (10) Å
*V* = 3037.1 (2) Å^3^
*Z* = 4
Mo *K*α radiation
μ = 0.08 mm^−1^
*T* = 296 (1) K
0.55 × 0.38 × 0.27 mm

#### Data collection

Rigaku R-AXIS RAPID diffractometer
Absorption correction: multi-scan (**ABSCOR**; Higashi, 1995 ▶) *T*_min_ = 0.938, *T*_max_ = 0.979
28664 measured reflections
3894 independent reflections
2600 reflections with *F* ^2^ > 2σ(*F* ^2^)
*R*_int_ = 0.049

#### Refinement

*R*[*F* ^2^ > 2σ(*F* ^2^)] = 0.042
*wR*(*F* ^2^) = 0.075
*S* = 1.00
3894 reflections
345 parameters
H-atom parameters constrained
Δρ_max_ = 0.15 e Å^−3^
Δρ_min_ = −0.16 e Å^−3^

### 

Data collection: *PROCESS-AUTO* (Rigaku/MSC, 2004 ▶); cell refinement: *PROCESS-AUTO*; data reduction: *CrystalStructure* (Rigaku/MSC, 2004 ▶); program(s) used to solve structure: *SIR97* (Altomare *et al.*, 1999 ▶); program(s) used to refine structure: *SHELXL97* (Sheldrick, 2008 ▶); molecular graphics: *PLATON* (Spek, 2003 ▶); software used to prepare material for publication: *CrystalStructure*.

## Supplementary Material

Crystal structure: contains datablocks global, I. DOI: 10.1107/S1600536808032959/is2332sup1.cif

Structure factors: contains datablocks I. DOI: 10.1107/S1600536808032959/is2332Isup2.hkl

Additional supplementary materials:  crystallographic information; 3D view; checkCIF report

## Figures and Tables

**Table 1:** Hydrogen-bond geometry (Å, °)

*D*—H⋯*A*	*D*—H	H⋯*A*	*D*⋯*A*	*D*—H⋯*A*
O1—H101⋯O4^i^	0.82	2.05	2.869 (2)	174
C19—H193⋯O1	0.96	2.45	3.127 (3)	127
C22—H222⋯O3	0.97	2.53	3.181 (3)	124
C32—H323⋯O1^ii^	0.96	2.59	3.363 (5)	137

Symmetry codes: (i) 
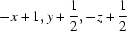
; (ii) 
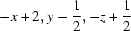
.

**Fig. 1.**
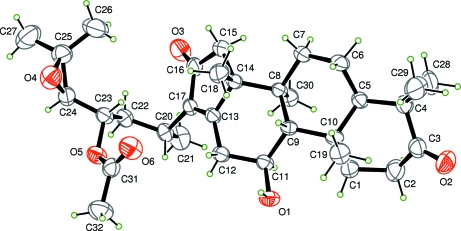
The molecular structure of (I), with atom labels, showing 40% probability displacement ellipsoids.

## supplementary crystallographic information

### Comment

Dry rhizome of Alisma orientale (Sam) Juzep (Alismataceae) is a traditional Chinese medicine, which was used as a diuretic in the treatment of oliguresis and edema. Phytochemical investigations of the species resulted in the isolation of a series of protostane-type triterpenes with a side chain connected to C-17, and oxygenated at C-3, 11, 16, 23, 24 and 25 (Yoshikawa *et al.*, 1993; Nakajima *et al.*, 1994). Some triterpenes from this plant showed a relaxant effect on the contraction of isolated aortic or bladder smooth muscles (Yoshikawa *et al.*, 1997). To investigate the bioactive natural products from this plant, we investigated its triterpene constituents, which led to the isolation of the title compound, alisol C 23-acetate, (I). Its structure was elucidated by spectroscopic analysis, and confirmed by a single-crystal X-ray diffraction analysis.

The relative stereochemistry of (I) has been determined (Fig. 1). The molecule contains three six-membered rings and one five-membered ring. Rings A (C1—C5/C10) and B (C5—C9/C10) adopt slightly distorted half-chair conformations owing to the ketone group at C3, and ring C (C8/C9/C11–14) exhibits a chair conformation. Ring D (C13—C17) adopts a plane conformation owing to the presence of the ketone group at C16. All rings are *trans*-fused. There is an intermolecular hydrogen bond between the hydroxy and epoxy groups.

### Experimental

Dried powder (5.0 kg) of the rhizone of Alisma orientale was extracted three times with 95% EtOH at room temperature. The solvent was removed by evaporation at reduced pressure and the residue was fractioned with chloroform. The residue of the chloroform fraction was subjected to column chromatography on silica gel, and eluted with petroleum ether-EtOH mixture. The crude compound was purified by column chromatography on silica gel with an petroleum ether-acetone mixture and recrystallized from petroleum ether-acetone (2:1) to afford 140 mg of the pure title compound, (I). Crystals suitable for X-ray structure analysis were obtained by slow evaporation of a petroleum ether-acetone (2:1) solution at room temperature (m.p. 505–507 K). ^13^C NMR (100 MHz, CDCl_3_): δ 31.1(C1), 33.7(C2), 218.6(C3), 47.1(C4) 48.6(C5), 20.2(C6), 35.0(C7), 40.3(C8), 49.0(C9), 37.1(C10), 69.8(C11) 35.8(C12), 176.3(C13), 49.7(C14), 45.9(C15), 207.2(C16), 138.2(C17), 23.2(C18), 25.7(C19), 26.9(C20), 20.3(C21) 35.3(C22), 71.9(C23), 64.9(C24), 58.7(C25), 20.0(C26), 24.8(C27), 29.7(C28), 19.5(C29), 23.2(C30), 169.6(C31), 21.5(C32).

### Refinement

All of the H atoms were placed in calculated positions (C—H = 0.96–0.98 Å and O—H = 0.82 Å) and allowed to ride on their parent atoms with *U*_iso_(H) = 1.2*U*_eq_(C) and 1.5*U*_eq_(O). In the absence of significant anomalous dispersion effects, Friedel pairs were averaged.

### Crystal data

**Table:**

C_32_H_48_O_6_	*F*(000) = 1152.00
*M**_r_* = 528.73	*D*_x_ = 1.156 Mg m^−^^3^
Orthorhombic, *P*2_1_2_1_2_1_	Mo *K*α radiation, λ = 0.71075 Å
Hall symbol: P 2ac 2ab	Cell parameters from 17721 reflections
*a* = 7.6879 (3) Å	θ = 3.0–27.4°
*b* = 14.6555 (6) Å	µ = 0.08 mm^−^^1^
*c* = 26.9557 (10) Å	*T* = 296 K
*V* = 3037.1 (2) Å^3^	Chunk, colorless
*Z* = 4	0.55 × 0.38 × 0.27 mm

### Data collection

**Table:**

Rigaku R-AXIS RAPID diffractometer	2600 reflections with *F*^2^ > 2σ(*F*^2^)
Detector resolution: 10.00 pixels mm^-1^	*R*_int_ = 0.049
ω scans	θ_max_ = 27.4°
Absorption correction: multi-scan (*ABSCOR*; Higashi, 1995)	*h* = −9→8
*T*_min_ = 0.938, *T*_max_ = 0.979	*k* = −18→18
28664 measured reflections	*l* = −34→34
3894 independent reflections	

### Refinement

**Table:**

Refinement on *F*^2^	H-atom parameters constrained
*R*[*F*^2^ > 2σ(*F*^2^)] = 0.042	*w* = 1/[σ^2^(*F*_o_^2^) + (0.003*P*)^2^ + 1.21*P*] where *P* = (*F*_o_^2^ + 2*F*_c_^2^)/3
*wR*(*F*^2^) = 0.075	(Δ/σ)_max_ < 0.001
*S* = 1.00	Δρ_max_ = 0.15 e Å^−^^3^
3894 reflections	Δρ_min_ = −0.16 e Å^−^^3^
345 parameters	Extinction correction: SHELXL97 (Sheldrick, 2008)
0 restraints	Extinction coefficient: 0.0061 (2)

### Special details

**Table:**

Geometry. ENTER SPECIAL DETAILS OF THE MOLECULAR GEOMETRY
Refinement. Refinement using reflections with *F*^2^ > 2.0 σ(*F*^2^). The weighted *R*-factor(*wR*), goodness of fit (*S*) and *R*-factor (gt) are based on *F*, with *F* set to zero for negative *F*. The threshold expression of *F*^2^ > 2.0 σ(*F*^2^) is used only for calculating *R*-factor (gt).

### Fractional atomic coordinates and isotropic or equivalent isotropic displacement parameters (Å^2^)

**Table:**

	*x*	*y*	*z*	*U*_iso_*/*U*_eq_	
O1	0.9079 (2)	0.36198 (12)	0.17123 (6)	0.0536 (5)	
O2	0.8415 (3)	0.73734 (14)	0.08083 (8)	0.0762 (6)	
O3	0.3371 (3)	0.04538 (13)	0.05351 (8)	0.0774 (7)	
O4	0.2703 (3)	−0.09965 (16)	0.23760 (6)	0.0743 (6)	
O5	0.6588 (2)	−0.03947 (12)	0.21762 (6)	0.0567 (5)	
O6	0.5884 (3)	0.10029 (14)	0.24578 (8)	0.0734 (6)	
C1	0.8778 (3)	0.49351 (19)	0.08259 (11)	0.0522 (7)	
C2	0.9137 (4)	0.5859 (2)	0.05699 (12)	0.0729 (9)	
C3	0.7907 (4)	0.6613 (2)	0.06907 (10)	0.0574 (8)	
C4	0.5994 (4)	0.63909 (18)	0.06307 (11)	0.0564 (8)	
C5	0.5682 (3)	0.53516 (18)	0.07159 (10)	0.0454 (6)	
C6	0.3822 (3)	0.51119 (18)	0.08536 (12)	0.0588 (8)	
C7	0.3453 (3)	0.41005 (18)	0.07801 (11)	0.0552 (7)	
C8	0.4999 (3)	0.34560 (17)	0.08954 (9)	0.0412 (6)	
C9	0.6367 (3)	0.39218 (17)	0.12400 (8)	0.0383 (5)	
C10	0.6982 (3)	0.49128 (17)	0.10832 (9)	0.0414 (6)	
C11	0.7867 (3)	0.32593 (18)	0.13584 (9)	0.0441 (6)	
C12	0.7249 (3)	0.23271 (18)	0.15519 (10)	0.0479 (6)	
C13	0.5777 (3)	0.19359 (17)	0.12627 (9)	0.0422 (6)	
C14	0.4269 (3)	0.25806 (17)	0.11658 (9)	0.0437 (6)	
C15	0.3048 (3)	0.19951 (18)	0.08388 (11)	0.0565 (7)	
C16	0.3938 (4)	0.10850 (19)	0.07820 (10)	0.0535 (7)	
C17	0.5575 (3)	0.11018 (17)	0.10672 (9)	0.0433 (6)	
C18	0.3370 (4)	0.2781 (2)	0.16659 (10)	0.0608 (8)	
C19	0.7123 (4)	0.54712 (18)	0.15655 (9)	0.0567 (7)	
C20	0.6764 (4)	0.02966 (18)	0.11109 (9)	0.0502 (7)	
C21	0.7635 (5)	0.0067 (2)	0.06118 (11)	0.0788 (10)	
C22	0.5824 (4)	−0.05448 (18)	0.13151 (9)	0.0509 (7)	
C23	0.5108 (3)	−0.0416 (2)	0.18363 (9)	0.0500 (6)	
C24	0.3985 (4)	−0.1196 (2)	0.19966 (10)	0.0554 (7)	
C25	0.2119 (4)	−0.1263 (2)	0.18803 (11)	0.0661 (8)	
C26	0.1196 (4)	−0.0528 (2)	0.15994 (12)	0.1002 (13)	
C27	0.1282 (5)	−0.2187 (2)	0.18659 (13)	0.1025 (13)	
C28	0.5589 (5)	0.6623 (2)	0.00834 (12)	0.0990 (14)	
C29	0.4887 (4)	0.7023 (2)	0.09565 (16)	0.0909 (12)	
C30	0.5868 (3)	0.31638 (18)	0.04010 (9)	0.0518 (7)	
C31	0.6825 (4)	0.0351 (2)	0.24614 (10)	0.0560 (7)	
C32	0.8436 (4)	0.0251 (2)	0.27699 (11)	0.0789 (10)	
H5	0.5897	0.5058	0.0395	0.055*	
H9	0.5763	0.4008	0.1557	0.046*	
H11	0.9672	0.4828	0.1073	0.063*	
H12	0.8817	0.4456	0.0578	0.063*	
H20	0.7687	0.0458	0.1346	0.060*	
H21	1.0296	0.6054	0.0665	0.088*	
H22	0.9098	0.5760	0.0214	0.088*	
H23	0.4457	0.0158	0.1857	0.060*	
H24	0.4588	−0.1781	0.2034	0.066*	
H61	0.3630	0.5267	0.1199	0.071*	
H62	0.3035	0.5462	0.0647	0.071*	
H71	0.2493	0.3933	0.0995	0.066*	
H72	0.3118	0.4008	0.0437	0.066*	
H101	0.8586	0.3692	0.1980	0.080*	
H111	0.8505	0.3152	0.1049	0.053*	
H121	0.8218	0.1904	0.1537	0.057*	
H122	0.6880	0.2399	0.1894	0.057*	
H151	0.1928	0.1920	0.0999	0.068*	
H152	0.2887	0.2280	0.0517	0.068*	
H181	0.3036	0.2216	0.1820	0.073*	
H182	0.2354	0.3148	0.1610	0.073*	
H183	0.4160	0.3103	0.1879	0.073*	
H191	0.5990	0.5537	0.1711	0.068*	
H192	0.7592	0.6063	0.1492	0.068*	
H193	0.7876	0.5161	0.1794	0.068*	
H211	0.6757	−0.0070	0.0370	0.095*	
H212	0.8309	0.0580	0.0502	0.095*	
H213	0.8383	−0.0452	0.0653	0.095*	
H221	0.6638	−0.1050	0.1320	0.061*	
H222	0.4863	−0.0689	0.1095	0.061*	
H261	−0.0015	−0.0531	0.1686	0.120*	
H262	0.1318	−0.0635	0.1250	0.120*	
H263	0.1693	0.0053	0.1682	0.120*	
H271	0.1980	−0.2612	0.2050	0.123*	
H272	0.0144	−0.2152	0.2011	0.123*	
H273	0.1188	−0.2387	0.1528	0.123*	
H281	0.6332	0.6272	−0.0130	0.119*	
H282	0.4396	0.6481	0.0013	0.119*	
H283	0.5788	0.7262	0.0027	0.119*	
H291	0.3677	0.6921	0.0888	0.109*	
H292	0.5174	0.7647	0.0885	0.109*	
H293	0.5117	0.6897	0.1300	0.109*	
H301	0.6758	0.2721	0.0468	0.062*	
H302	0.5009	0.2902	0.0186	0.062*	
H303	0.6377	0.3688	0.0244	0.062*	
H321	0.9431	0.0432	0.2579	0.095*	
H322	0.8345	0.0631	0.3059	0.095*	
H323	0.8563	−0.0374	0.2870	0.095*	

### Atomic displacement parameters (Å^2^)

**Table:**

	*U*^11^	*U*^22^	*U*^33^	*U*^12^	*U*^13^	*U*^23^
O1	0.0422 (10)	0.0596 (12)	0.0590 (10)	−0.0007 (10)	−0.0170 (9)	−0.0020 (9)
O2	0.0834 (17)	0.0582 (13)	0.0871 (15)	−0.0209 (13)	−0.0096 (14)	0.0027 (12)
O3	0.1001 (18)	0.0547 (13)	0.0775 (14)	−0.0029 (14)	−0.0350 (14)	−0.0082 (11)
O4	0.0846 (16)	0.0903 (16)	0.0479 (11)	−0.0117 (15)	0.0047 (11)	0.0012 (11)
O5	0.0595 (12)	0.0544 (11)	0.0561 (10)	0.0068 (11)	−0.0144 (10)	−0.0020 (10)
O6	0.0839 (17)	0.0616 (13)	0.0748 (13)	0.0081 (14)	−0.0117 (13)	−0.0101 (11)
C1	0.0437 (16)	0.0561 (17)	0.0568 (16)	0.0026 (14)	0.0059 (14)	0.0033 (14)
C2	0.061 (2)	0.063 (2)	0.095 (2)	−0.0008 (18)	0.0171 (19)	0.0168 (18)
C3	0.065 (2)	0.0525 (18)	0.0545 (16)	−0.0043 (17)	−0.0056 (16)	0.0074 (14)
C4	0.0584 (19)	0.0385 (15)	0.0722 (19)	0.0011 (15)	−0.0149 (16)	−0.0003 (13)
C5	0.0454 (15)	0.0389 (14)	0.0521 (14)	0.0014 (13)	−0.0067 (13)	0.0007 (12)
C6	0.0414 (16)	0.0457 (17)	0.089 (2)	0.0075 (14)	−0.0104 (16)	−0.0016 (15)
C7	0.0442 (16)	0.0464 (16)	0.0749 (18)	0.0018 (14)	−0.0155 (15)	0.0071 (14)
C8	0.0408 (14)	0.0399 (14)	0.0429 (13)	0.0047 (12)	−0.0072 (12)	0.0006 (11)
C9	0.0354 (13)	0.0416 (13)	0.0379 (12)	0.0037 (11)	−0.0012 (11)	−0.0031 (11)
C10	0.0379 (14)	0.0437 (14)	0.0425 (13)	0.0027 (12)	−0.0013 (11)	−0.0031 (11)
C11	0.0399 (14)	0.0481 (15)	0.0444 (13)	0.0040 (13)	−0.0078 (12)	−0.0026 (12)
C12	0.0458 (17)	0.0465 (15)	0.0512 (15)	0.0041 (13)	−0.0114 (13)	0.0024 (12)
C13	0.0435 (15)	0.0456 (15)	0.0376 (12)	0.0049 (13)	−0.0049 (12)	0.0039 (11)
C14	0.0395 (15)	0.0417 (14)	0.0498 (14)	0.0034 (13)	−0.0071 (12)	0.0008 (12)
C15	0.0501 (17)	0.0460 (16)	0.0733 (18)	−0.0006 (15)	−0.0183 (16)	0.0033 (14)
C16	0.065 (2)	0.0484 (16)	0.0476 (14)	−0.0067 (16)	−0.0113 (14)	0.0028 (13)
C17	0.0511 (16)	0.0391 (14)	0.0398 (12)	0.0030 (13)	−0.0031 (12)	0.0030 (11)
C18	0.0528 (18)	0.0592 (18)	0.0703 (18)	0.0033 (16)	0.0117 (16)	0.0061 (15)
C19	0.068 (2)	0.0534 (17)	0.0489 (15)	−0.0040 (17)	−0.0024 (15)	−0.0080 (13)
C20	0.0600 (18)	0.0441 (15)	0.0466 (14)	0.0091 (15)	0.0019 (14)	−0.0029 (12)
C21	0.093 (2)	0.079 (2)	0.0644 (19)	0.017 (2)	0.0239 (19)	−0.0034 (17)
C22	0.0620 (18)	0.0430 (15)	0.0477 (14)	0.0081 (15)	−0.0016 (14)	−0.0019 (12)
C23	0.0515 (16)	0.0487 (16)	0.0499 (15)	0.0031 (15)	−0.0092 (13)	0.0038 (13)
C24	0.0614 (19)	0.0520 (17)	0.0528 (15)	−0.0005 (16)	−0.0031 (14)	0.0060 (13)
C25	0.060 (2)	0.084 (2)	0.0537 (17)	−0.0043 (19)	−0.0031 (15)	0.0031 (16)
C26	0.063 (2)	0.148 (3)	0.089 (2)	0.021 (2)	−0.005 (2)	0.027 (2)
C27	0.089 (3)	0.116 (3)	0.103 (2)	−0.042 (2)	−0.004 (2)	−0.005 (2)
C28	0.135 (3)	0.054 (2)	0.108 (2)	−0.020 (2)	−0.067 (2)	0.0293 (19)
C29	0.069 (2)	0.0450 (19)	0.158 (3)	0.0084 (18)	0.004 (2)	−0.016 (2)
C30	0.0636 (19)	0.0502 (16)	0.0417 (13)	−0.0022 (16)	−0.0072 (13)	−0.0032 (12)
C31	0.062 (2)	0.0603 (19)	0.0460 (15)	−0.0077 (18)	−0.0019 (15)	0.0061 (15)
C32	0.078 (2)	0.090 (2)	0.069 (2)	−0.001 (2)	−0.0240 (19)	−0.0026 (18)

### Geometric parameters (Å, °)

**Table:**

O1—C11	1.434 (3)	C2—H22	0.970
O2—C3	1.223 (3)	C5—H5	0.980
O3—C16	1.220 (3)	C6—H61	0.970
O4—C24	1.450 (3)	C6—H62	0.970
O4—C25	1.463 (3)	C7—H71	0.970
O5—C23	1.461 (3)	C7—H72	0.970
O5—C31	1.349 (3)	C9—H9	0.980
O6—C31	1.198 (3)	C11—H111	0.980
C1—C2	1.544 (4)	C12—H121	0.970
C1—C10	1.545 (3)	C12—H122	0.970
C2—C3	1.491 (4)	C15—H151	0.970
C3—C4	1.515 (4)	C15—H152	0.970
C4—C5	1.559 (3)	C18—H181	0.960
C4—C28	1.546 (4)	C18—H182	0.960
C4—C29	1.534 (4)	C18—H183	0.960
C5—C6	1.518 (3)	C19—H191	0.960
C5—C10	1.547 (3)	C19—H192	0.960
C6—C7	1.522 (3)	C19—H193	0.960
C7—C8	1.550 (3)	C20—H20	0.980
C8—C9	1.560 (3)	C21—H211	0.960
C8—C14	1.579 (3)	C21—H212	0.960
C8—C30	1.551 (3)	C21—H213	0.960
C9—C10	1.585 (3)	C22—H221	0.970
C9—C11	1.541 (3)	C22—H222	0.970
C10—C19	1.540 (3)	C23—H23	0.980
C11—C12	1.538 (3)	C24—H24	0.980
C12—C13	1.489 (3)	C26—H261	0.960
C13—C14	1.518 (3)	C26—H262	0.960
C13—C17	1.340 (3)	C26—H263	0.960
C14—C15	1.547 (3)	C27—H271	0.960
C14—C18	1.543 (3)	C27—H272	0.960
C15—C16	1.507 (3)	C27—H273	0.960
C16—C17	1.475 (4)	C28—H281	0.960
C17—C20	1.497 (3)	C28—H282	0.960
C20—C21	1.540 (4)	C28—H283	0.960
C20—C22	1.532 (3)	C29—H291	0.960
C22—C23	1.521 (3)	C29—H292	0.960
C23—C24	1.497 (4)	C29—H293	0.960
C24—C25	1.472 (4)	C30—H301	0.960
C25—C26	1.496 (5)	C30—H302	0.960
C25—C27	1.499 (5)	C30—H303	0.960
C31—C32	1.499 (4)	C32—H321	0.960
C1—H11	0.970	C32—H322	0.960
C1—H12	0.970	C32—H323	0.960
C2—H21	0.970		
			
C24—O4—C25	60.70 (19)	H61—C6—H62	109.5
C23—O5—C31	118.7 (2)	C6—C7—H71	108.0
C2—C1—C10	112.3 (2)	C6—C7—H72	108.0
C1—C2—C3	116.0 (2)	C8—C7—H71	108.0
O2—C3—C2	122.0 (3)	C8—C7—H72	108.0
O2—C3—C4	122.3 (2)	H71—C7—H72	109.5
C2—C3—C4	115.7 (2)	C8—C9—H9	104.8
C3—C4—C5	110.1 (2)	C10—C9—H9	104.8
C3—C4—C28	104.5 (2)	C11—C9—H9	104.8
C3—C4—C29	110.3 (2)	O1—C11—H111	107.5
C5—C4—C28	109.0 (2)	C9—C11—H111	107.5
C5—C4—C29	114.9 (2)	C12—C11—H111	107.5
C28—C4—C29	107.6 (2)	C11—C12—H121	108.4
C4—C5—C6	114.0 (2)	C11—C12—H122	108.4
C4—C5—C10	113.6 (2)	C13—C12—H121	108.4
C6—C5—C10	110.9 (2)	C13—C12—H122	108.4
C5—C6—C7	111.7 (2)	H121—C12—H122	109.5
C6—C7—C8	115.1 (2)	C14—C15—H151	110.4
C7—C8—C9	111.7 (2)	C14—C15—H152	110.4
C7—C8—C14	108.4 (2)	C16—C15—H151	110.4
C7—C8—C30	109.1 (2)	C16—C15—H152	110.4
C9—C8—C14	108.68 (19)	H151—C15—H152	109.5
C9—C8—C30	110.0 (2)	C14—C18—H181	109.5
C14—C8—C30	109.00 (19)	C14—C18—H182	109.5
C8—C9—C10	116.32 (19)	C14—C18—H183	109.5
C8—C9—C11	110.6 (2)	H181—C18—H182	109.5
C10—C9—C11	114.1 (2)	H181—C18—H183	109.5
C1—C10—C5	106.3 (2)	H182—C18—H183	109.5
C1—C10—C9	113.9 (2)	C10—C19—H191	109.5
C1—C10—C19	107.8 (2)	C10—C19—H192	109.5
C5—C10—C9	111.0 (2)	C10—C19—H193	109.5
C5—C10—C19	111.4 (2)	H191—C19—H192	109.5
C9—C10—C19	106.43 (19)	H191—C19—H193	109.5
O1—C11—C9	113.1 (2)	H192—C19—H193	109.5
O1—C11—C12	107.60 (19)	C17—C20—H20	107.6
C9—C11—C12	113.5 (2)	C21—C20—H20	107.6
C11—C12—C13	113.5 (2)	C22—C20—H20	107.6
C12—C13—C14	115.5 (2)	C20—C21—H211	109.5
C12—C13—C17	130.1 (2)	C20—C21—H212	109.5
C14—C13—C17	114.3 (2)	C20—C21—H213	109.5
C8—C14—C13	108.3 (2)	H211—C21—H212	109.5
C8—C14—C15	113.8 (2)	H211—C21—H213	109.5
C8—C14—C18	114.1 (2)	H212—C21—H213	109.5
C13—C14—C15	102.5 (2)	C20—C22—H221	108.4
C13—C14—C18	108.1 (2)	C20—C22—H222	108.4
C15—C14—C18	109.4 (2)	C23—C22—H221	108.4
C14—C15—C16	105.9 (2)	C23—C22—H222	108.4
O3—C16—C15	124.4 (2)	H221—C22—H222	109.5
O3—C16—C17	127.0 (2)	O5—C23—H23	110.1
C15—C16—C17	108.6 (2)	C22—C23—H23	110.1
C13—C17—C16	108.6 (2)	C24—C23—H23	110.1
C13—C17—C20	128.1 (2)	O4—C24—H24	115.1
C16—C17—C20	123.3 (2)	C23—C24—H24	115.1
C17—C20—C21	111.7 (2)	C25—C24—H24	115.1
C17—C20—C22	112.0 (2)	C25—C26—H261	109.5
C21—C20—C22	110.1 (2)	C25—C26—H262	109.5
C20—C22—C23	113.7 (2)	C25—C26—H263	109.5
O5—C23—C22	107.5 (2)	H261—C26—H262	109.5
O5—C23—C24	106.5 (2)	H261—C26—H263	109.5
C22—C23—C24	112.4 (2)	H262—C26—H263	109.5
O4—C24—C23	116.2 (2)	C25—C27—H271	109.5
O4—C24—C25	60.08 (19)	C25—C27—H272	109.5
C23—C24—C25	123.5 (2)	C25—C27—H273	109.5
O4—C25—C24	59.22 (18)	H271—C27—H272	109.5
O4—C25—C26	114.6 (2)	H271—C27—H273	109.5
O4—C25—C27	113.4 (2)	H272—C27—H273	109.5
C24—C25—C26	121.4 (3)	C4—C28—H281	109.5
C24—C25—C27	119.0 (3)	C4—C28—H282	109.5
C26—C25—C27	115.7 (3)	C4—C28—H283	109.5
O5—C31—O6	124.0 (2)	H281—C28—H282	109.5
O5—C31—C32	110.4 (2)	H281—C28—H283	109.5
O6—C31—C32	125.6 (2)	H282—C28—H283	109.5
C11—O1—H101	109.3	C4—C29—H291	109.5
C2—C1—H11	108.8	C4—C29—H292	109.5
C2—C1—H12	108.8	C4—C29—H293	109.5
C10—C1—H11	108.8	H291—C29—H292	109.5
C10—C1—H12	108.8	H291—C29—H293	109.5
H11—C1—H12	109.5	H292—C29—H293	109.5
C1—C2—H21	107.8	C8—C30—H301	109.5
C1—C2—H22	107.8	C8—C30—H302	109.5
C3—C2—H21	107.8	C8—C30—H303	109.5
C3—C2—H22	107.8	H301—C30—H302	109.5
H21—C2—H22	109.5	H301—C30—H303	109.5
C4—C5—H5	105.8	H302—C30—H303	109.5
C6—C5—H5	105.8	C31—C32—H321	109.5
C10—C5—H5	105.8	C31—C32—H322	109.5
C5—C6—H61	108.9	C31—C32—H323	109.5
C5—C6—H62	108.9	H321—C32—H322	109.5
C7—C6—H61	108.9	H321—C32—H323	109.5
C7—C6—H62	108.9	H322—C32—H323	109.5
			
C24—O4—C25—C26	−113.3 (3)	C30—C8—C14—C18	179.9 (2)
C24—O4—C25—C27	110.9 (3)	C8—C9—C10—C1	−101.6 (2)
C25—O4—C24—C23	115.3 (2)	C8—C9—C10—C5	18.5 (2)
C23—O5—C31—O6	0.7 (4)	C8—C9—C10—C19	139.8 (2)
C23—O5—C31—C32	−178.2 (2)	C8—C9—C11—O1	−174.33 (18)
C31—O5—C23—C22	121.3 (2)	C8—C9—C11—C12	−51.4 (2)
C31—O5—C23—C24	−118.1 (2)	C10—C9—C11—O1	52.2 (2)
C2—C1—C10—C5	43.8 (2)	C10—C9—C11—C12	175.14 (19)
C2—C1—C10—C9	166.4 (2)	C11—C9—C10—C1	29.1 (2)
C2—C1—C10—C19	−75.8 (2)	C11—C9—C10—C5	149.2 (2)
C10—C1—C2—C3	11.3 (3)	C11—C9—C10—C19	−89.5 (2)
C1—C2—C3—O2	131.8 (3)	O1—C11—C12—C13	170.9 (2)
C1—C2—C3—C4	−51.5 (3)	C9—C11—C12—C13	45.0 (2)
O2—C3—C4—C5	−154.7 (2)	C11—C12—C13—C14	−48.6 (3)
O2—C3—C4—C28	88.4 (3)	C11—C12—C13—C17	130.5 (2)
O2—C3—C4—C29	−26.9 (3)	C12—C13—C14—C8	56.2 (2)
C2—C3—C4—C5	28.5 (3)	C12—C13—C14—C15	176.7 (2)
C2—C3—C4—C28	−88.3 (2)	C12—C13—C14—C18	−67.8 (2)
C2—C3—C4—C29	156.3 (2)	C12—C13—C17—C16	−175.6 (2)
C3—C4—C5—C6	158.5 (2)	C12—C13—C17—C20	3.8 (4)
C3—C4—C5—C10	30.1 (3)	C14—C13—C17—C16	3.5 (3)
C28—C4—C5—C6	−87.5 (3)	C14—C13—C17—C20	−177.1 (2)
C28—C4—C5—C10	144.1 (2)	C17—C13—C14—C8	−123.0 (2)
C29—C4—C5—C6	33.2 (3)	C17—C13—C14—C15	−2.5 (2)
C29—C4—C5—C10	−95.2 (3)	C17—C13—C14—C18	112.9 (2)
C4—C5—C6—C7	162.7 (2)	C8—C14—C15—C16	117.1 (2)
C4—C5—C10—C1	−67.5 (2)	C13—C14—C15—C16	0.4 (2)
C4—C5—C10—C9	168.1 (2)	C18—C14—C15—C16	−114.0 (2)
C4—C5—C10—C19	49.7 (2)	C14—C15—C16—O3	−178.0 (2)
C6—C5—C10—C1	162.6 (2)	C14—C15—C16—C17	1.5 (2)
C6—C5—C10—C9	38.1 (2)	O3—C16—C17—C13	176.4 (2)
C6—C5—C10—C19	−80.3 (2)	O3—C16—C17—C20	−3.0 (4)
C10—C5—C6—C7	−67.5 (3)	C15—C16—C17—C13	−3.1 (3)
C5—C6—C7—C8	35.1 (3)	C15—C16—C17—C20	177.5 (2)
C6—C7—C8—C9	20.9 (3)	C13—C17—C20—C21	−111.2 (3)
C6—C7—C8—C14	140.6 (2)	C13—C17—C20—C22	124.8 (2)
C6—C7—C8—C30	−100.9 (2)	C16—C17—C20—C21	68.0 (3)
C7—C8—C9—C10	−49.0 (2)	C16—C17—C20—C22	−55.9 (3)
C7—C8—C9—C11	178.7 (2)	C17—C20—C22—C23	−62.1 (2)
C7—C8—C14—C13	178.1 (2)	C21—C20—C22—C23	173.1 (2)
C7—C8—C14—C15	64.9 (2)	C20—C22—C23—O5	−71.3 (2)
C7—C8—C14—C18	−61.5 (2)	C20—C22—C23—C24	171.8 (2)
C9—C8—C14—C13	−60.3 (2)	O5—C23—C24—O4	87.1 (2)
C9—C8—C14—C15	−173.5 (2)	O5—C23—C24—C25	157.2 (2)
C9—C8—C14—C18	60.0 (2)	C22—C23—C24—O4	−155.4 (2)
C14—C8—C9—C10	−168.5 (2)	C22—C23—C24—C25	−85.3 (3)
C14—C8—C9—C11	59.2 (2)	O4—C24—C25—C26	101.7 (3)
C30—C8—C9—C10	72.3 (2)	O4—C24—C25—C27	−101.4 (3)
C30—C8—C9—C11	−60.1 (2)	C23—C24—C25—O4	−103.3 (2)
C30—C8—C14—C13	59.5 (2)	C23—C24—C25—C26	−1.6 (4)
C30—C8—C14—C15	−53.7 (2)	C23—C24—C25—C27	155.3 (2)

### Hydrogen-bond geometry (Å, °)

**Table:**

*D*—H···*A*	*D*—H	H···*A*	*D*···*A*	*D*—H···*A*
O1—H101···O4^i^	0.82	2.05	2.869 (2)	174
C19—H193···O1	0.96	2.45	3.127 (3)	127
C22—H222···O3	0.97	2.53	3.181 (3)	124
C32—H323···O1^ii^	0.96	2.59	3.363 (5)	137

Symmetry codes: (i) −*x*+1, *y*+1/2, −*z*+1/2; (ii) −*x*+2, *y*−1/2, −*z*+1/2.

## References

[bb1] Altomare, A., Burla, M. C., Camalli, M., Cascarano, G. L., Giacovazzo, C., Guagliardi, A., Moliterni, A. G. G., Polidori, G. & Spagna, R. (1999). *J. Appl. Cryst.* **32**, 115–119.

[bb2] Higashi, T. (1995). *ABSCOR* Rigaku Corporation, Tokyo, Japan.

[bb3] Nakajima, Y., Satoh, Y., Katsumata, M., Tsujiyama, K., Ida, Y. & Shoh, J. (1994). *Phytochemistry*, **36**, 119–127.

[bb4] Rigaku/MSC (2004). *PROCESS-AUTO* and *CrystalStructure* Rigaku/MSC, The Woodlands, Texas, USA.

[bb5] Sheldrick, G. M. (2008). *Acta Cryst.* A**64**, 112–122. 10.1107/S0108767307043930 18156677

[bb6] Spek, A. L. (2003). *J. Appl. Cryst.* **36**, 7–13.

[bb7] Yoshikawa, M., Hatakeyama, S., Tanaka, N., Fukuda, Y., Yamahara, J. & Murakami, N. (1993). *Chem. Pharm. Bull.* **41**, 1948–1954. 10.1248/cpb.41.1194 8370117

[bb8] Yoshikawa, M., Murakami, T., Ikebata, A., Ishikado, A., Murakami, N., Yamahara, J. & Matsuda, H. (1997). *Chem. Pharm. Bull.* **45**, 756–758. 10.1248/cpb.45.1186 9246753

